# Vaccine research in cognitive impairment and dementia: a bibliometric analysis and future outlook (2000–2025)

**DOI:** 10.3389/fneur.2025.1662314

**Published:** 2025-11-12

**Authors:** Zhongli Wang, Xiaojia Xue, Hua Zhang, Jipeng Liu, Shoujie Dai, Haiping Duan, Shixue Li

**Affiliations:** 1 Centre for Health Management and Policy Research, School of Public Health, Cheeloo College of Medicine, Shandong University, Jinan, China; 2 NHC Key Lab of Health Economics and Policy Research, Shandong University, Jinan, China; 3 Qingdao Municipal Center for Disease Control and Prevention, Qingdao, Shandong, China; 4 Pingdu Center for Disease Control and Prevention, Qingdao, Shandong, China

**Keywords:** cognitive impairment, dementia, vaccine, immunotherapy, bibliometric analysis

## Abstract

**Background:**

Vaccine research has shifted from a purely anti-infective tool to a promising therapeutic and preventive strategy against cognitive impairment (CI) and dementia. Yet the knowledge domain linking vaccine to neurodegeneration has never been systematically mapped.

**Methods:**

We retrieved English-language articles and reviews on vaccine and CI/dementia in Web of Science and PubMed (2000–2025). After rigorous screening, 1,395 publications were analyzed with Excel, VOSviewer, CiteSpace, Scimago Graphica and Charticulator to chart output trajectories, collaborative networks, influential journals, high-impact papers, keyword evolution and co-citation clusters.

**Results:**

Annual publications rose nearly 10-fold, peaking at >90 papers in 2022. The United States dominated output and collaboration, flanked by an increasingly interconnected European core and a fast growing Asia-Pacific node. Four institutional clusters were identified, with the UC system, NYU and Harvard leading. Author co-authorship revealed a dense hub around Cribbs, Agadjanyan and Ghochikyan, while recent entrants from China and Europe diversified the landscape. Keyword, timeline and burst analyses showed a thematic shift from β-amyloid vaccine studies (2000–2008) to passive immunotherapy and biomarker-guided translation (2009–2016), and, most recently (2017–2024), to multivalent vaccine engineering, immunosenescence. Co-citation clusters tracked the field’s progression from plaque-centred paradigms to oligomer-targeted, multi-pathway approaches and highlighted emerging interest in innate immunity and infection-mediated neuroinflammation.

**Conclusion:**

Vaccine research in CI/dementia has matured into a multidisciplinary, prevention-oriented enterprise. Future priorities include (i) multi-epitope or mRNA-based vaccines that address amyloid, tau and inflammatory cascades; (ii) strategies to overcome immunosenescence for earlier, prophylactic immunization; (iii) mechanistic and interventional studies to validate the neuroprotective signals seen with routine adult vaccines. These directions will be pivotal for translating immunological insights into effective, population-level protection against dementia.

## 1 Introduction

With the accelerating global aging process, cognitive impairment (CI) and dementia have emerged as significant public health challenges (1). In 2019, approximately 57.4 million individuals worldwide were living with dementia, and this figure is projected to rise to 153 million by 2050 (2). These conditions profoundly impact memory, cognitive function, and daily living abilities, imposing a substantial socioeconomic burden (3). In 2015, the global economic cost attributable to dementia reached $818 billion, equivalent to 1.1% of global GDP (4). Currently, dementia remains incurable, and existing pharmacological interventions offer limited therapeutic efficacy, highlighting an urgent need for effective prevention and disease-modifying strategies (5).

Traditionally, vaccines stimulate immune responses to prevent infectious diseases. Recently, researchers have explored vaccines for neurodegenerative disorders, aiming to utilize immunotherapy to prevent or treat Alzheimer’s disease (AD) and related dementias. For instance, in 1999, Schenk et al. (6) first reported that a vaccine targeting beta-amyloid (Aβ) effectively cleared amyloid plaques and improved pathological outcomes in mouse models. This breakthrough led to the first clinical trial of an AD vaccine. However, in a 2002 phase II trial, approximately 6% of participants experienced severe adverse effects, notably meningoencephalitis, prompting the trial’s termination (7). Despite initial setbacks, subsequent follow-up studies revealed partial pathological improvements in patients, thereby validating the potential of immunotherapy (8). Researchers subsequently revised their strategies, shifting to passive immunization or optimizing vaccine designs to enhance safety profiles. Recent passive immunization approaches, such as mAbs therapies, such as aducanumab and lecanemab, demonstrated efficacy in amyloid plaque removal and slowing cognitive decline in clinical trials (9, 10). In 2021, aducanumab, the first immunotherapy targeting amyloid, received conditional approval from the U.S. FDA, sparking extensive discussion (11). These advances underscore the viability of immunological interventions targeting abnormal brain proteins and have rekindled enthusiasm for active immunization research.

Beyond therapeutic applications, epidemiological studies indicate unexpected benefits of routine vaccines in dementia prevention. Multiple large-scale cohort studies have reported significantly reduced risks of AD or dementia among older adults receiving routine vaccines (12). For example, a U.S. study involving nearly one million elderly individuals found a roughly 40% lower risk of AD within 4 years among those vaccinated against influenza compared to unvaccinated counterparts (13). Subsequent investigations confirmed similar associations with Tdap, herpes zoster (HZ) and pneumococcal vaccines, demonstrating risk reductions of approximately 30, 25 and 27%, respectively (14). Notably, a 2024 UK-based study of 200,000 older adults showed that recipients of the recombinant HZ vaccine had significantly lower dementia incidence over 6 years than recipients of the live-attenuated vaccine, extending dementia-free survival by about 17% (≈ 164 dementia-free days) (15). In addition, treatment with the Bacillus Calmette–Guérin (BCG) vaccine, primarily used for non-muscle-invasive bladder cancer, was linked to an ≈ 20% reduced risk of AD and related dementias during 15 years of follow-up (16). Mechanistically, such associations are biologically plausible via two non-exclusive pathways. First, suppression of viral reactivations after zoster vaccination may attenuate systemic-to-central neuroinflammation and help maintain blood–brain barrier integrity (15). Second, vaccines and their adjuvants can reprogram immune tone in aging, through elements of trained innate immunity and Th1-skewing, potentially reducing pro-inflammatory microglial priming and improving handling of protein aggregates (17). Concordantly, vaccination dose–response patterns for lower incident dementia, consistent with fewer infection-triggered inflammatory episodes (18). Collectively, these findings suggest that vaccines may positively influence cognitive health through immune modulation, reduction of chronic inflammation or prevention of infections, while helping dispel misconceptions that vaccine harms cognition.

Given this context, research exploring CI/dementia and vaccine has attracted increasing attention over the past two decades, with rapid growth in literature spanning animal studies of pathological mechanisms, clinical vaccine trials, and epidemiological evaluations of population-based preventive effects (19), thereby forming an interdisciplinary knowledge domain. A systematic literature review in this field is warranted to elucidate the evolution and thematic trends of this research area. Bibliometric analysis, employing quantitative statistical and visualization methods, can effectively reveal the structure and dynamics of specific research fields. Numerous medical disciplines have utilized bibliometric analyses to identify research trends and predict future directions. For example, bibliometric studies on depression-dementia comorbidity have highlighted emerging themes like gut microbiota (20). However, to date, no bibliometric study has specifically addressed the intersection of CI/dementia and vaccine. This study aims to fill this research gap.

Using the Web of Science (WOS) and PubMed, this study systematically retrieved English-language literature published between 2000 and 2025 on CI/dementia and vaccine. Applying bibliometric methods, we quantitatively analyzed publication trends, geographic and institutional distribution, scholar collaboration networks, productive journals and highly-cited papers, keyword hotspots, thematic evolution, and co-citation clusters. By examining these factors, we aim to outline the developmental trajectory and research hotspots within this domain, providing valuable references for researchers and policymakers and suggesting directions for future research.

## 2 Materials and methods

### 2.1 Data acquisition and processing

This study systematically searched the WOS and PubMed databases on April 21, 2025, covering the period from January 1, 2000, to April 10, 2025. The following search strategy was used: (“vaccine*” OR “vaccin*”) AND (“Alzheimer” OR “Senile Dementia” OR “Primary Senile Degenerative Dementia” OR “Presenile Dementia”). In WOS, the search was conducted using Topic Search (TS), which covers titles, abstracts, author keywords, and Keywords Plus. In PubMed, the search was limited to Title and Abstract fields to enhance specificity. We identified 2,763 records in total (WOS, *n* = 1,607; PubMed, *n* = 1,156). Exports from both databases were appended and de-duplicated with a hierarchical procedure: primary matching by DOI, then PMID if DOI was missing, and finally a normalized combination of title, first author, and year using high-threshold fuzzy matching when identifiers were unavailable. This process removed 940 duplicate entries, leaving 1,823 unique records for screening.

**Figure 1:**
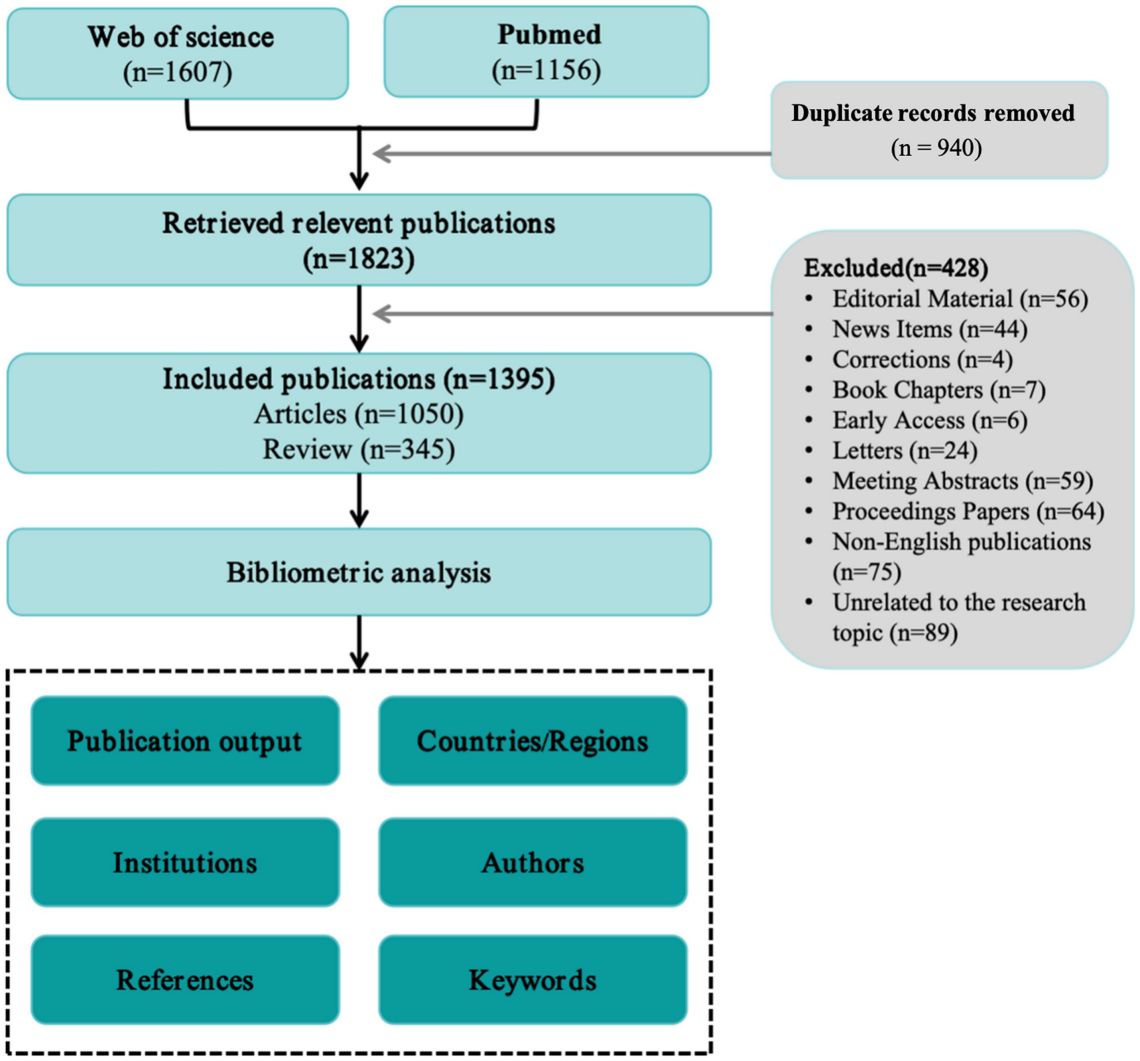
Flowchart for identifying and selecting publications.

Two reviewers (ZW and XX) independently screened titles/abstracts against prespecified eligibility criteria, restricting inclusion to English-language publications and document types article or review. Prior to analysis, we standardized author names, normalized journal and institution labels, harmonized keywords, and used the year of the version of record. A total of 428 records were excluded during screening: Editorial Material (*n* = 56), News Items (*n* = 44), Corrections (*n* = 4), Book Chapters (*n* = 7), Early Access (*n* = 6), Letters (*n* = 24), Meeting Abstracts (*n* = 59), Proceedings Papers (*n* = 64), non-English publications (*n* = 75), and studies unrelated to the topic (*n* = 89). And 1,395 publications were included in the final dataset. The selection process is summarized in Figure 1.

### 2.2 Bibliometric analysis and visualization

Bibliometric data were processed and visualized with Microsoft Excel 2021, VOSviewer v1.6.20, CiteSpace v6.4 R1, Scimago Graphica and Charticulator to provide a comprehensive portrait of the research domain. Microsoft Excel was used to tabulate annual publication counts from 2000 to 2025, thereby revealing long-term output trends. VOSviewer, developed by Martins et al. (21), was employed to generate maps of inter-country collaboration, co-authorship networks, institutional partnerships, temporal overlay visualizations, and keyword co-occurrence. Scimago Graphica depicted yearly outputs for the most productive countries, whereas Charticulator^1^ produced chord diagrams illustrating international collaboration patterns without the need for coding. CiteSpace, created by Chen et al. (22), was used to construct journal dual-map overlays, perform keyword and reference clustering, detect citation bursts, and visualize the resulting networks. The CiteSpace analysis was run with the following parameters: software version 6.4 R1; time span 2000–2025 with a one-year slice length; node selection based on the *g*-index (*k* = 25); Pathfinder network pruning; and labels displayed for 1% of the nodes.

## 3 Results

### 3.1 Overview of research trends

**Figure 2:**
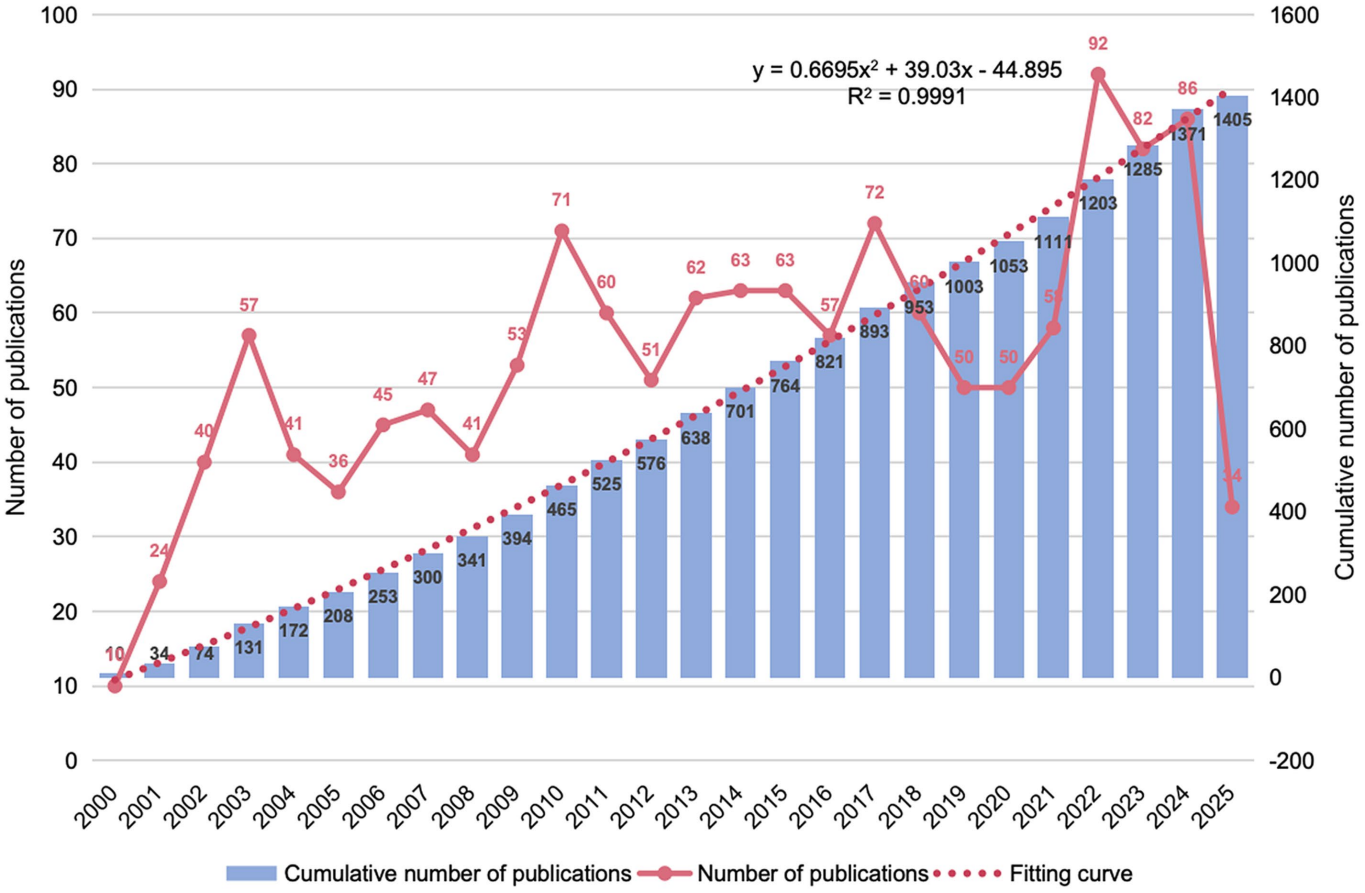
Annual and cumulative publication trends (2000–2025).

Research output on CI/dementia and vaccine has climbed steadily since 2000. Annual papers moved from single-digit levels to about 50 by the mid-2000s, rose sharply around 2010, and peaked at more than 90 in 2022. Although yearly counts eased modestly in 2023–2024, the cumulative total exceeded 1,400 publications by 2024, and a near-quadratic fit to the cumulative curve (*R*^2^ ≈ 0.999) in Figure 2 indicates that expansion is likely to continue; the lower tally registered so far for 2025 almost certainly reflects indexing delay rather than an actual decline.

### 3.2 Country analysis

**Figure 3:**
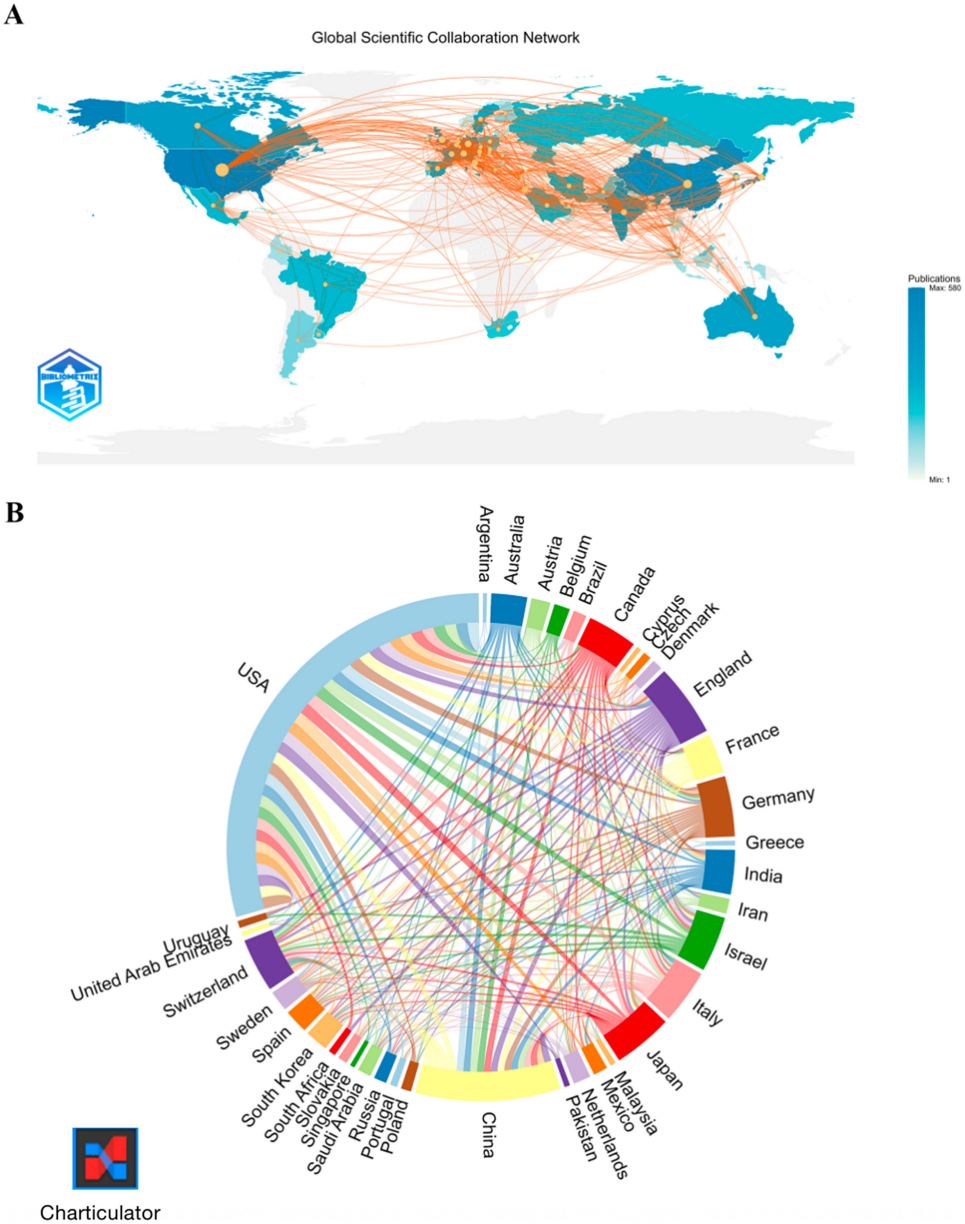
Global collaboration network. **(A)** Co-authorship map. **(B)** Collaboration chord diagram.

Among the top publishing countries (Table 1), the United States leads decisively with 478 papers, 37,818 citations and an *H*-index of 223, followed by China (154 papers) and Japan (63 papers); meanwhile, the United Kingdom, Italy, Canada, Germany and Switzerland pair moderate output with high citation influence (*H*-index ≥42), whereas India’s growing productivity is not yet matched by citation impact. The global collaboration map (Figure 3A) and chord diagram (Figure 3B) visualize this landscape: a hub-and-spoke network centred on the USA, a densely interconnected European core, and an expanding Asia-Pacific cluster that increasingly links to both North America and Europe.

**Table 1:** Top 10 countries in terms of publications.

Rank	Countries	Counts	Citations	*H*-index
1	USA	478	37,818	223
2	China	154	3,961	53
3	Japan	63	1,603	48
4	Israel	58	3,516	45
5	United Kingdom	56	5,692	87
6	Italy	55	3,057	62
7	India	46	766	33
8	Canada	45	4,187	74
9	Germany	37	2,032	50
10	Switzerland	34	2,604	42

### 3.3 Institutions analysis

**Figure 4:**
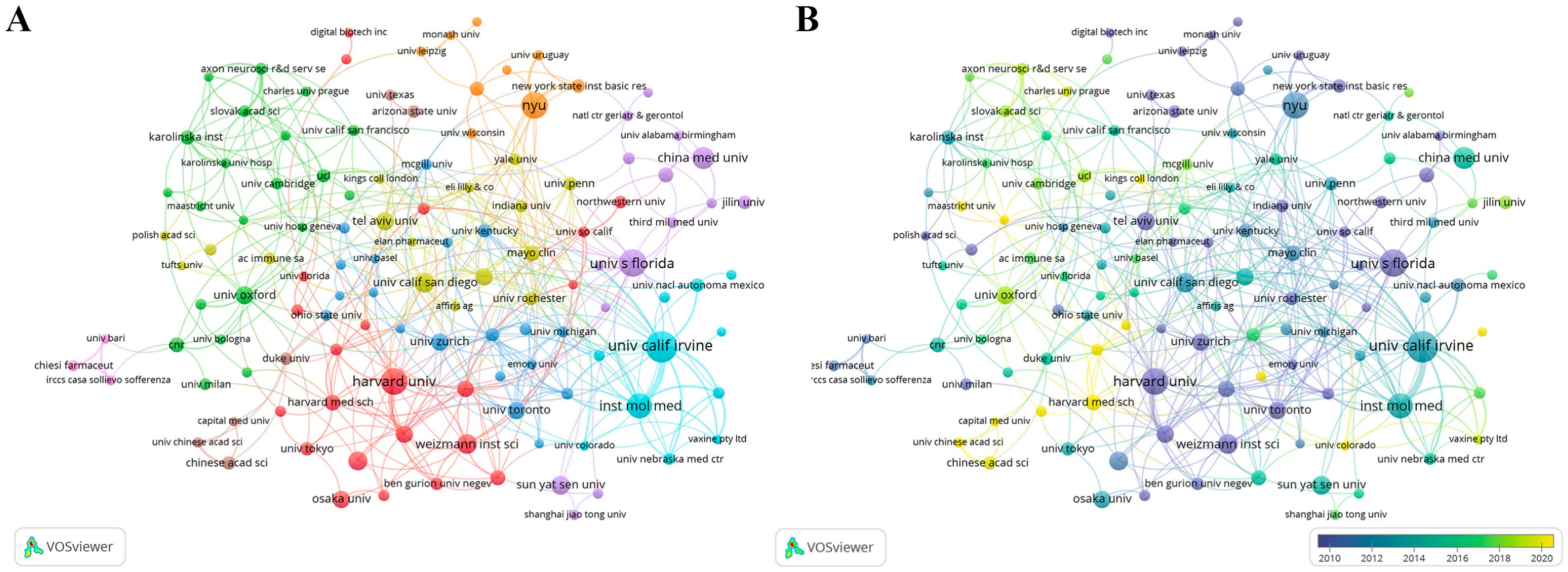
Institutional collaboration network. **(A)** Co-authorship clusters. **(B)** Temporal overlay of collaborations.

CI/dementia and vaccine research is spearheaded by U.S. institutions. The University of California system tops the list with 214 publications and an *H*-index of 74, followed by New York University and Harvard University (Table 2). Figure 4A shows four interlinked collaboration clusters. Harvard–Weizmann hub spanning North America and Israel; European grouping centred on Oxford, Cambridge and Karolinska; West-Coast network anchored by UC Irvine and the Institute of Molecular Medicine; and Florida-based cluster around the State University System of Florida. The time-overlay map (Figure 4B) indicates that early leaders such as Harvard, UC San Diego and Oxford have recently been joined by emerging hubs, including UC Irvine, NYU and several Chinese universities, signalling a broader, more globally distributed institutional landscape.

**Table 2:** Top 10 institutions in terms of publications.

Rank	Institutions	Counts	Citations	*H*-index
1	University of California System	214	37,068	74
2	New York University	119	19,448	50
3	Harvard University	115	27,023	45
4	State University System of Florida	114	10,864	40
5	University of California Irvine	114	10,929	39
6	University of South Florida	97	10,339	38
7	University of British Columbia	53	10,508	35
8	University of California Los Angeles	39	5,003	35
9	Harvard University Medical Affiliates	74	9,215	33
10	University of Texas System	68	7,973	32

### 3.4 Author analysis

**Figure 5:**
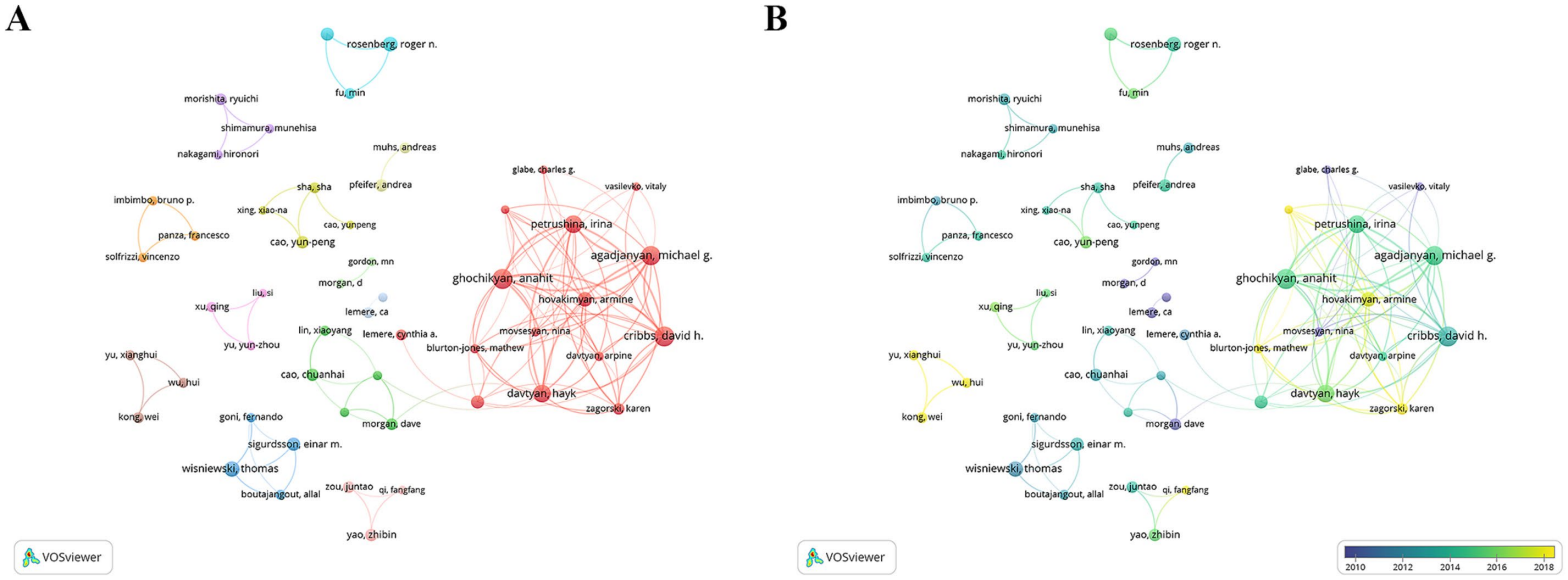
Author collaboration network. **(A)** Co-authorship clusters. **(B)** Temporal overlay of collaborations.

Leading contributors in CI/dementia and vaccine research are concentrated within a few tightly knit author teams. As summarized in Table 3, Cribbs DH heads the field with 38 publications (*H*-index 22), closely followed by Agadjanyan MG and Ghochikyan A (34 each), all three belonging to the same highly collaborative group that also includes Petrushina I. and Davtyan H. Additionally, Morgan D. (26 papers, 2,980 citations) and Lemere C. A. (21 papers, 1,431 citations) provide additional high-impact hubs, while Wisniewski T. and Masliah E. stand out for strong citation influence relative to output. The co-authorship map (Figure 5A) confirms a pronounced core-periphery structure: a dense red cluster centred on Cribbs–Agadjanyan–Ghochikyan dominates inter-author links, whereas smaller, more loosely connected clusters represent Japanese, Chinese and European teams. Temporal overlay colouring (Figure 5B) indicates that early pioneers such as Lemere and Morgan (blue-green nodes, 2010–2013) have been joined by the Cribbs-led consortium and several Chinese investigators after 2014 (yellow nodes), signalling both continuity of established collaborations and the gradual entry of new research groups into the network.

**Table 3:** Top 10 authors in terms of publications.

Rank	Author	Count	Citation	*H*-index
1	Cribbs D. H.	38	1,391	22
2	Agadjanyan M. G.	34	1,174	20
3	Ghochikyan A.	34	1,187	20
4	Petrushina I.	29	1,078	19
5	Morgan D.	26	2,980	19
6	Davtyan H.	26	973	19
7	Wisniewski T.	24	1,583	19
8	Lemere C. A.	21	1,431	17
9	Masliah E.	19	2,224	16
10	Schwartz M.	18	2,515	15

### 3.5 Journals analysis

**Figure 6:**
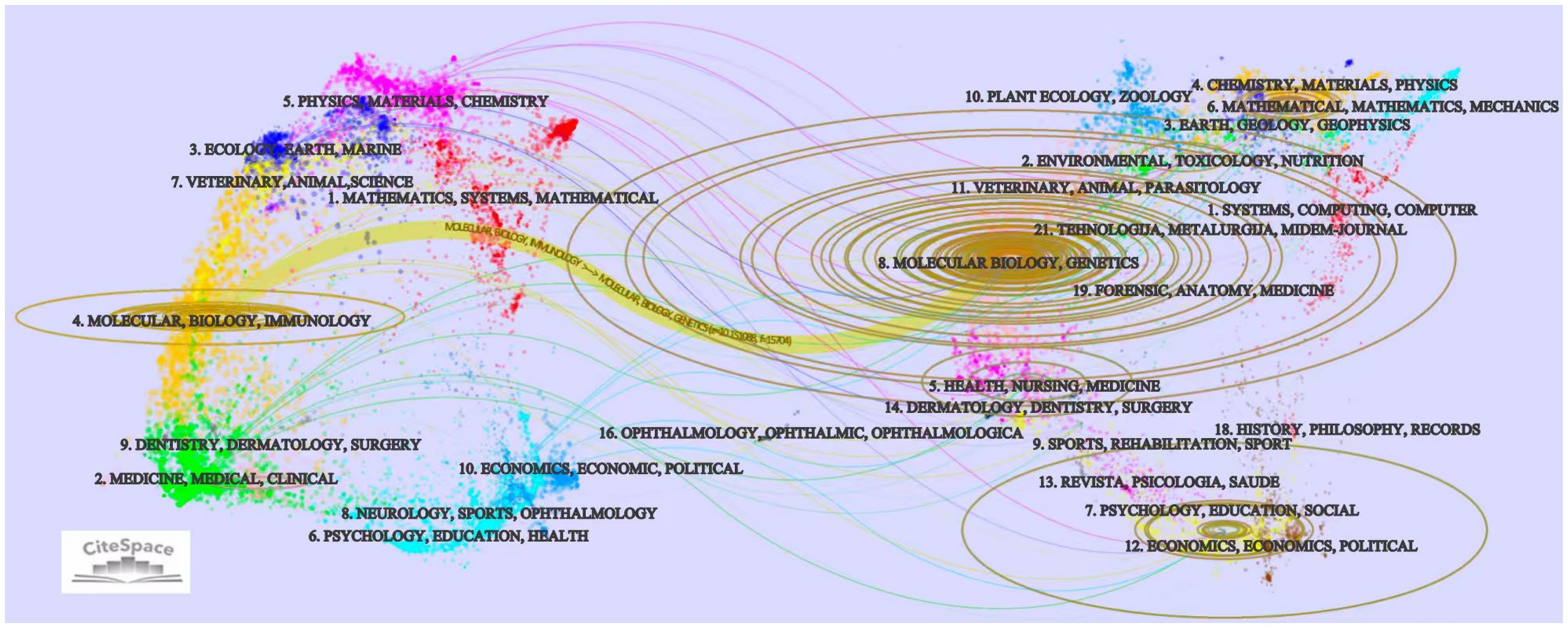
The journal dual-map overlay.

Research on CI/dementia and vaccine is channelled through a relatively small set of specialist and high-impact outlets (Table 4). The Journal of Alzheimer’s Disease leads with 56 papers, followed by Vaccine (*n* = 29) and PLoS One (*n* = 25). Higher-impact neuroscience titles, such as Journal of Neuroscience (*n* = 23) and Neurobiology of Disease (*n* = 18), attract substantial contributions, while Proceedings of the National Academy of Sciences of the United States of America offers the highest impact factor in the top tier (IF 9.1, Q1) despite a smaller count of 15 papers. The journal dual-map overlay (Figure 6) traces citations from “Medicine, Medical, Clinical” and “Molecular Biology, Immunology” sources on the left to “Molecular Biology, Genetics” and “Health, Nursing, Medicine” targets on the right, highlighting the translational linkage between basic immunology/neuroscience and clinical practice. Additional, thinner paths connect to psychology-education and social-science clusters, suggesting growing attention to behavioral and economic facets of vaccine in dementia.

**Table 4:** Top 10 journals in terms of publications.

Rank	Journals	Count	IF	JCR
1	Journal of Alzheimers Disease	56	3.1	Q2
2	Vaccine	29	3.5	Q2
3	PLoS One	25	2.6	Q2
4	Journal of Neuroscience	23	4.4	Q1
5	Journal of Neuroimmunology	22	2.5	Q3
6	Neurobiology of Aging	21	3.5	Q2
7	Current Alzheimer Research	19	1.9	Q3
8	Neurobiology of Disease	18	5.6	Q1
9	Scientific Reports	16	3.9	Q1
10	Proceedings of the National Academy of Sciences of the United States of America	15	9.1	Q1

### 3.6 Keywords analysis

**Figure 7:**
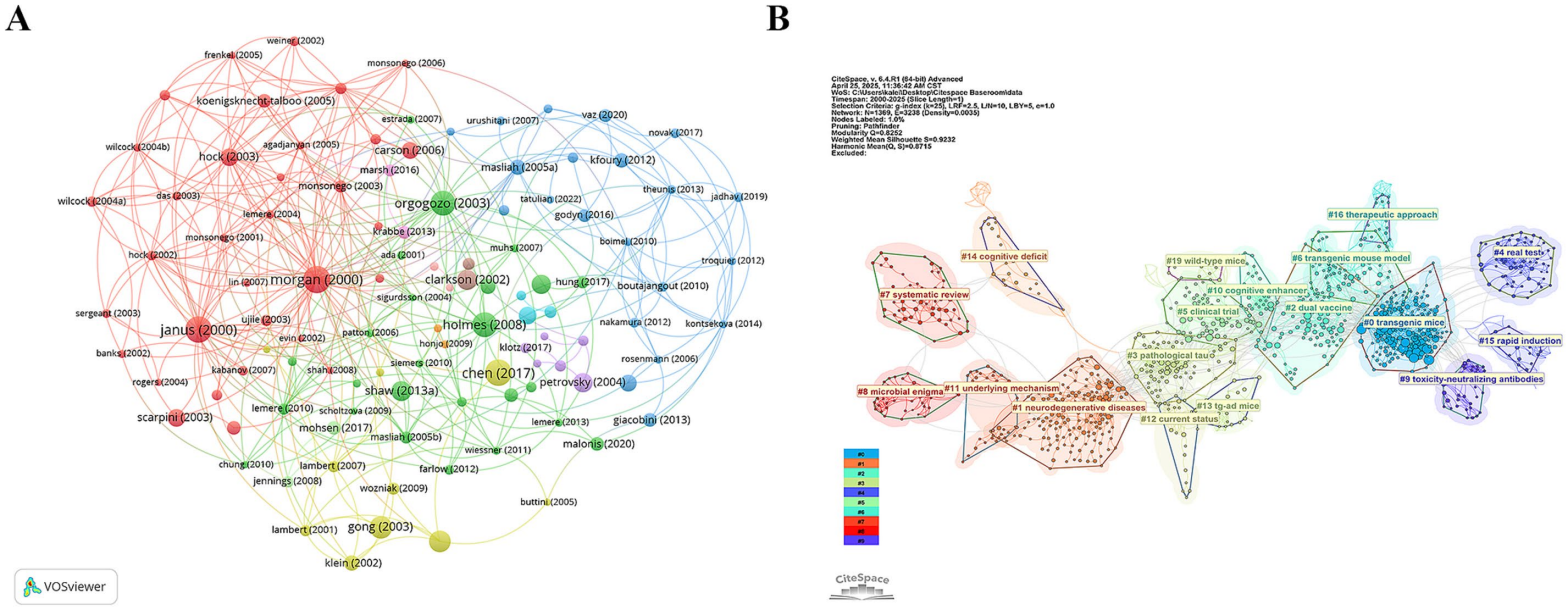
Mapping of references. **(A)** References co-occurrence network and **(B)** References-cluster timeline.

The keyword co-occurrence network derived from 1,395 publications (Figure 7A) clearly centred on an “Alzheimer’s disease-immune intervention” hub. Five high-degree keywords “Alzheimer’s disease,” “amyloid beta,” “mouse model,” “vaccine” and “immunization” (Table 5)—formed the structural backbone, linking experimental animal work to vaccine design. Bridging nodes such as “immune-response,” “antibody” and “inflammation” connected pathological and immunological sub-modules.

**Table 5:** The top 20 Keywords in terms of publications.

Rank	Keywords	Count
1	Alzheimer’s disease	906
2	amyloid beta	505
3	mouse model	484
4	vaccine	410
5	immunization	301
6	immunotherapy	279
7	transgenic mice	247
8	peptide	194
9	antibody	182
10	pathology	181
11	central-nervous-system	145
12	protein	126
13	brain	118
14	immune-response	113
15	tau protein	102
16	dementia	100
17	neurodegeneration	92
18	*in-vivo*	85
19	inflammation	85
20	passive immunization	76

Figure 7B arrays 12 clusters along a 2000–2025 timeline. Phase I (2000–2007) is dominated by antigen-discovery work in #5 transgenic mice, #6 amyloid beta, and #7 tau pathology; Phase II (2008–2016) shifts toward clinical translation with #1 monoclonal antibodies and biomarker-driven #4 cerebrospinal fluid; and Phase III (2017–2025) introduces implementation and engineering themes—#3 cost-effectiveness analysis, #8 innate immunity, #9 single-chain variable fragment, #10 amyloid-beta peptide, and #11 aggregation inhibitors. The backbone #0 Alzheimer’s disease runs throughout. These clusters depict a progression from mechanistic antigen studies to passive immunotherapies with biomarker monitoring, and finally to population-level, cost-conscious, bio-engineered vaccine strategies.

**Figure 8:**
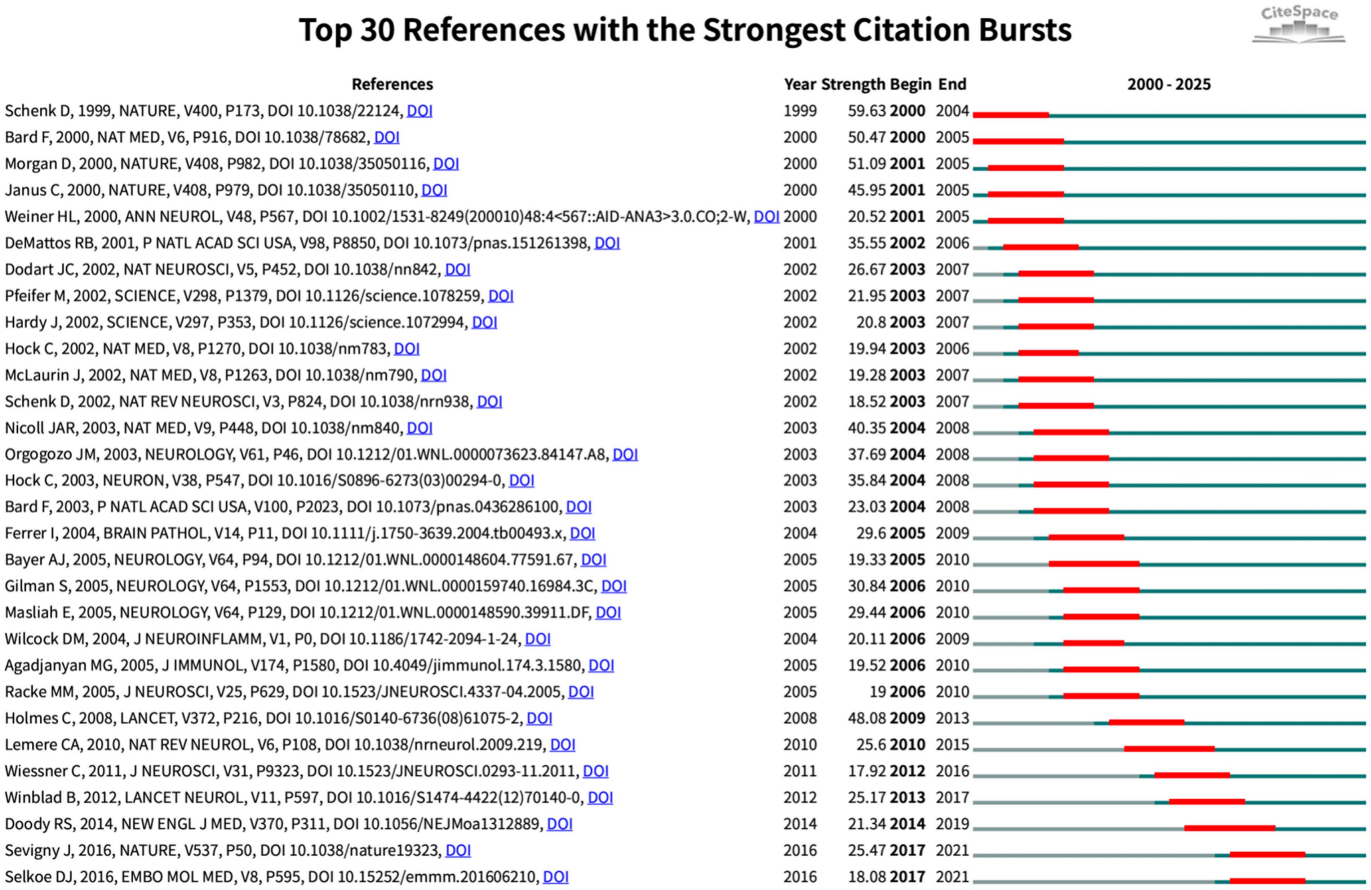
Top 30 references with strongest citation bursts (red bars indicate burst periods; blue timeline indicates 2000–2025).

Burst analysis (Figure 8) further highlighted the dynamic evolution of the field. Early bursts in “pathology” (2001–2004) mirrored the pathological validation phase, whereas the surge in “immunotherapy” (2014–2019) marked maturation of therapeutic antibody approaches. Recent keyword bursts for “Parkinson’s disease” (2016–2025) and for “infection,” “influenza vaccination” and “risk factors” (2020–2025) indicate a growing emphasis on epidemiological studies exploring how routine vaccines and infectious exposures shape CI/dementia risk. The evidence traces a trajectory from mechanistic, β-amyloid-centred vaccine studies to a contemporary, prevention-oriented agenda that leverages large cohorts and cross-disease perspectives.

### 3.7 Reference co-citation analysis

**Figure 9:**
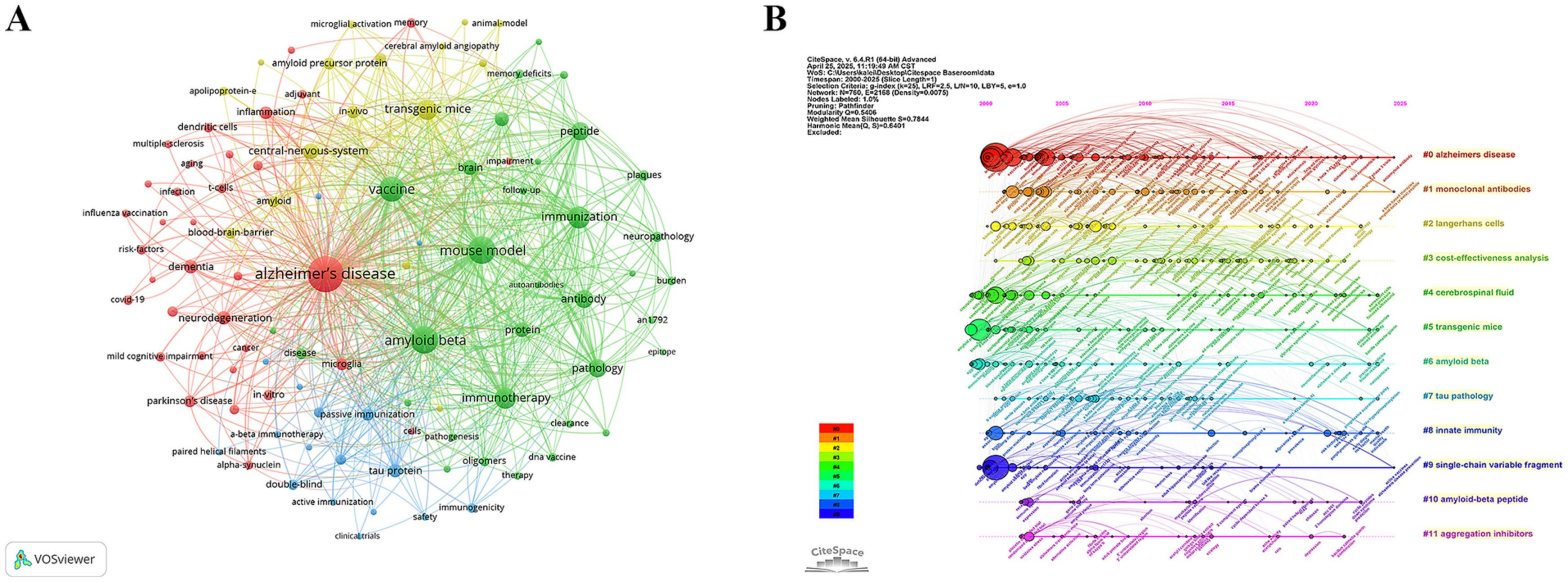
Mapping of keywords. **(A)** Keyword co-occurrence network. **(B)** Keyword-cluster timeline.

**Figure 10:**
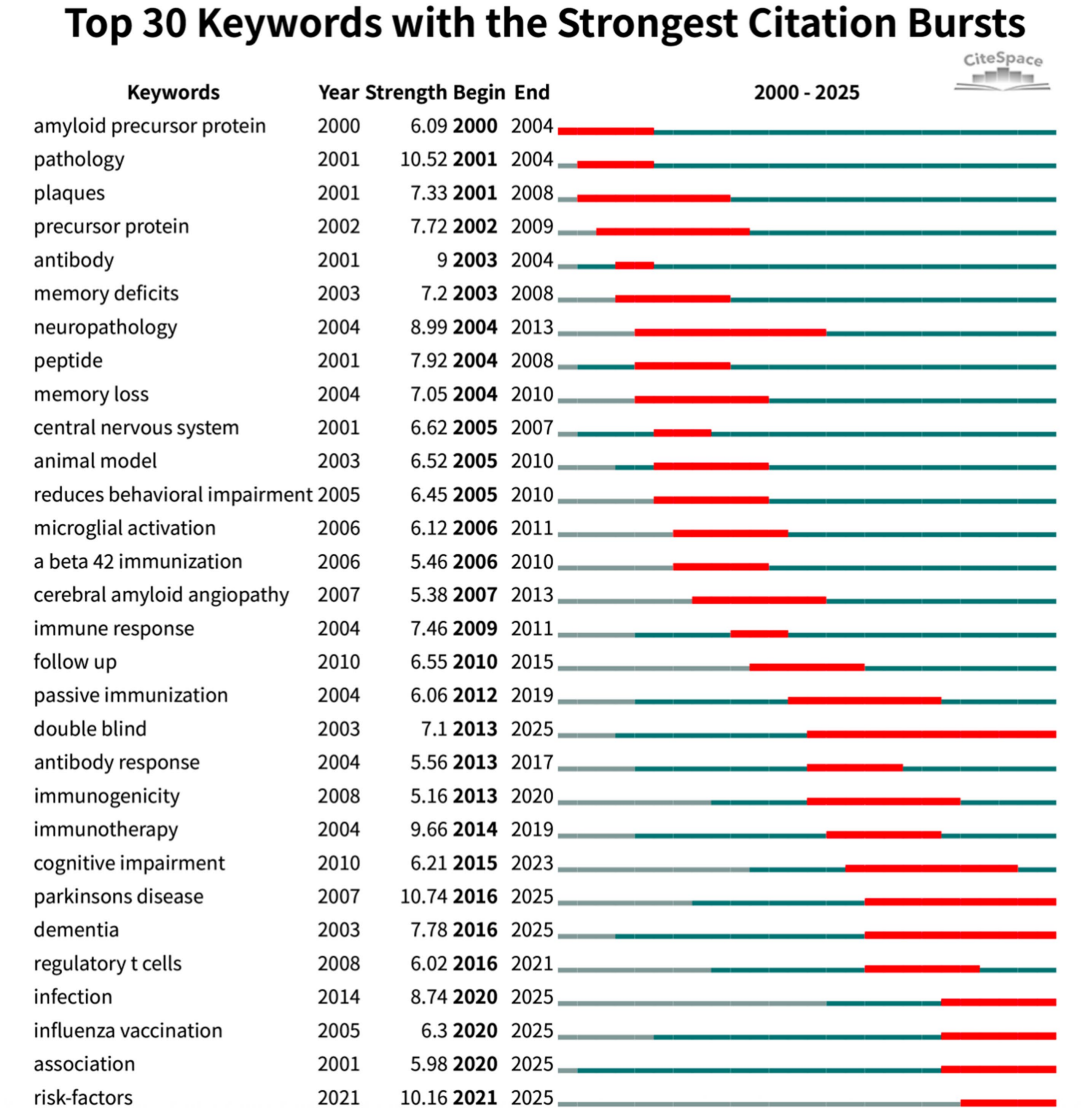
Top 30 keywords with strongest citation bursts (red bars indicate burst periods; blue timeline indicates 2000–2025).

Figure 9A visualises their interconnections, revealing three time-stratified co-citation modules: an early red cluster of foundational Aβ-vaccine studies (2000–2005), a mid-period green cluster bridging animal data to first-in-human trials (2003–2010), and a blue cluster (post-2015) focused on multivalent designs. CiteSpace clustering (Figure 9B; *Q* = 0.85, *S* = 0.92) sharpens these into themes such as “transgenic mice,” “pathological tau,” “dual vaccine” and “toxicity-neutralizing antibodies” highlighting the shift from single-target strategies to combination and safety-oriented approaches. Citation-burst detection (Figure 10) corroborates this progression, marking three attention waves: foundational immunization work (2000–2005), clinical translation and safety debates (2009–2014), and a recent surge (2017–2021) centred on antibody engineering and immunosenescence.

The 10 most co-cited papers (8, 23–31) (Table 6) fall into three tightly knit thematic clusters that together trace the conceptual evolution of CI/dementia and vaccine research.

**Table 6:** The top 10 co-cited references.

Rank	Author (year), Country	Citations	Study type	Main conclusion	Research significance
1	Holmes C. (2008), UK	1,138	Follow-up of RCT	AN1792 Aβ₄₂ immunization cleared amyloid plaques but did not halt cognitive decline or neurodegeneration	Demonstrated that removing amyloid plaques alone is insufficient to stop dementia progression, reshaping AD therapy strategies
2	Blennow K. (2003), Sweden	979	Review	Identified CSF biomarkers as promising tools for early detection of incipient Alzheimer’s in MCI patients	Pioneered use of biomarkers for early Alzheimer’s diagnosis, enabling earlier intervention in cognitive decline
3	Gong Y. (2003), USA	917	Experimental study	Discovered soluble Aβ oligomers accumulate in AD brains, linking them to reversible memory loss and validating them as therapy targets	Provided key evidence for the “oligomer hypothesis,” shifting AD research toward targeting small toxic Aβ aggregates
4	Klein W. L. (2001), USA	894	Perspective/Review	Proposed that small Aβ oligomers are the critical neurotoxin in AD; suggested neutralizing these toxins could improve vaccine efficacy	Reframed the amyloid hypothesis by highlighting oligomers as culprits, influencing new therapeutic approaches
5	Shaw A. C. (2013), USA	780	Review	Showed immunosenescence: aging causes chronic innate immune activation and impaired responses, contributing to inflammation-linked diseases like AD	Linked age-related immune changes to dementia, underscoring the role of inflammation in cognitive aging and AD pathology
6	Clarkson T. W. (2002), USA	763	Review	Examined human exposure to mercury and its neurotoxicology; highlighted widespread low-level exposure and unknown risks to brain health	Raised awareness of environmental toxins’ impact on cognition, informing public health policies on heavy metals and brain health
7	Petrovsky N. (2004), Australia	728	Review	Summarized vaccine adjuvants and stressed the urgent need for safer, more effective adjuvants to induce protective immunity	Guided development of new vaccine adjuvants, crucial for designing Alzheimer’s immunotherapies with minimal side effects
8	Hynd M. R. (2004), Australia	716	Review	Presented evidence that glutamate excitotoxicity via NMDA receptors contributes to AD neurodegeneration; excessive NMDA activity may drive neuron loss in AD	Highlighted a non-amyloid mechanism in AD, supporting use of NMDA antagonists and research into glutamatergic targets
9	Scarpini E. (2003), Italy	641	Review	Overview of AD treatments: cholinesterase inhibitors provide modest symptomatic benefit, and emerging therapies aim to modify disease	Provided a comprehensive reference on AD therapy status, guiding clinicians and highlighting new research directions for disease-modifying treatments
10	Hock C. (2003), Switzerland	635	Clinical trial analysis	In a halted Aβ vaccine trial, AD patients who developed anti-amyloid antibodies had significantly slower cognitive decline than those who did not	Offered clinical proof-of-concept that amyloid immunotherapy can benefit patients, energizing further vaccine and antibody trials in Alzheimer’s despite earlier setbacks

#### 3.7.1 Immunotherapy shifting focus from plaques to oligomers

Early vaccine studies demonstrated that eliminating fibrillar amyloid deposits alone was insufficient for clinical improvement (32). For example, the AN1792 follow-up study by Holmes et al. (8) reported successful amyloid clearance without any cognitive benefits. Meanwhile, laboratory and theoretical research (24, 25) revealed that soluble Aβ oligomers, rather than mature plaques, are primarily responsible for synaptic toxicity. Supporting this notion, a small clinical series by Hock et al. (31) showed that patients who generated a robust anti-Aβ antibody response experienced slower cognitive decline. These findings collectively prompted a shift in vaccine development toward targeting toxic oligomers and enhancing antibody specificity and function, rather than focusing solely on plaque reduction.

#### 3.7.2 Establishing the basis for very early intervention

Blennow and Hampel (23), the only diagnostic study among these key papers, identified cerebrospinal fluid biomarkers as reliable indicators of prodromal AD in patients with mild CI. The widespread adoption of these biomarkers provided the field with a practical tool for early detection, thereby paving the way for preventive vaccination strategies prior to the onset of overt dementia symptoms.

#### 3.7.3 Expanding the therapeutic framework

A third group of studies emphasized the importance of addressing additional pathological mechanisms beyond amyloid. Shaw et al. (26) examined how age-related innate immune dysfunction impairs vaccine responsiveness. Clarkson et al. (27) drew attention to the neurotoxic effects of chronic low-level mercury exposure, a frequently overlooked environmental factor. Hynd et al. (29) explored the role of glutamate-mediated excitotoxicity in neuronal damage. Petrovsky and Aguilar (28) critically reviewed the limitations of traditional alum-based adjuvants and proposed novel adjuvant strategies aimed at promoting a more effective Th1-skewed immune response. Finally, Scarpini et al. (30) integrated symptomatic treatment options with emerging disease-modifying approaches. Collectively, these studies conceptualize dementia as a multifactorial condition and support the use of combinatorial strategies, such as improved adjuvants, anti-inflammatory or neuroprotective co-treatments, and mitigation of environmental risks, to enhance both the effectiveness and durability of vaccine-based interventions.

## 4 Discussion

### 4.1 Overall trend analysis

The domain of dementia and vaccine has expanded dramatically over the past two decades, reflecting the urgent need for novel interventions against cognitive decline in an aging world (33, 34). Annual publication outputs grew from only a handful in 2000 to nearly 100 by 2022, with a near-exponential cumulative increase (*R*^2^ ≈ 0.999) indicating sustained momentum (35). This surge corresponds to rising scientific and public interest following key breakthroughs and challenges in the field. Early proof-of-concept studies demonstrated that immune approaches could modulate Alzheimer’s pathology (36), sparking optimism, while setbacks such as the 2002 AN1792 trial’s neuroinflammatory complications tempered expectations (37). Nonetheless, continued refinements in immunotherapy, as evidenced by the recent approvals of monoclonal antibodies that showed amyloid clearance and modest cognitive benefit (38, 39). In parallel, epidemiological discoveries that routine vaccinations (influenza, tetanus/diphtheria/pertussis, shingles, pneumococcal) are associated with significantly lower dementia risk in older adults (40–43) have opened a complementary prevention-oriented research avenue. These developments have not only driven publication counts but also broadened the disciplinary landscape of the field, bringing together experts in neurology, immunology, geriatrics, public health and even health economics. Our bibliometric analysis confirmed a globally distributed and collaborative research network led by the United States, with increasing contributions from Europe, China, and other regions, indicating that progress in this domain is a worldwide endeavor. Such international collaboration is visualized by a “hub-and-spoke” network centred on the US and a tightly-knit European cluster, now augmented by emerging Asian-Pacific partnerships. Taken together, the overall trend underscores an evolving, interdisciplinary field that has rapidly matured from preliminary trials to a multifaceted research enterprise spanning molecular mechanisms to population-level studies.

### 4.2 Key research hotspots

Early hotspots in this field coalesced around immunotherapy for AD, particularly vaccines targeting the Aβ protein. Pioneering work in transgenic mice first established that Aβ vaccination could reduce plaque burden (36, 44) leading to clinical trials of Aβ vaccines. The first human vaccine trial (AN1792) proved the principle of amyloid clearance but revealed critical safety issues when ~6% of patients developed meningoencephalitis (35). Follow-up analyses showed that although immunized patients had substantial plaque removal at autopsy, their neurodegeneration and clinical decline continued unabated (8). These findings, echoed by others, shifted the research focus toward targeting toxic Aβ oligomers rather than insoluble plaques. Laboratory and theoretical studies demonstrated that soluble oligomeric Aβ species are the chief mediators of synaptic toxicity in AD (45–47). Correspondingly, second-generation vaccine strategies and passive immunotherapies have aimed at neutralizing oligomers or preventing their aggregation (48). Antibodies developed in recent years preferentially target aggregated Aβ and have shown the ability to clear plaques and modestly slow cognitive decline in trials (49). These advances in passive immunization have reinvigorated active vaccine research, with designs now emphasizing safety and specificity—such as using shorter Aβ fragments, avoiding T-cell epitopes that triggered past inflammation, and employing novel adjuvants (37). Another prominent hotspot is tau protein vaccines and combination therapies. As the limits of Aβ-centric approaches became evident (50, 51), researchers broadened their scope to other pathology. Several groups are developing vaccines against pathological tau proteins to curb neurofibrillary tangle formation, and exploring multi-target vaccines that might simultaneously address Aβ, tau, or other pathogenic proteins. Indeed, co-citation analysis revealed clusters labeled “pathological tau” and “dual vaccine,” indicating significant efforts to tackle multiple hallmarks of neurodegeneration in tandem (52–54). Alongside these specific targets, neuroinflammation and immunomodulation have emerged as key themes. Many studies highlight the role of chronic inflammation in CI and the potential for vaccines to modulate the immune system’s behavior in the aging brain (55, 56). Research on innate immunity and immunosenescence is particularly salient. Aging-related declines in immune function can dampen vaccine efficacy, so recent hotspots include strategies to overcome poor immune responses in the elderly (57). In summary, current research hotspots span from molecular immunotherapy to vaccinology and gerontology, all converging on the goal of altering the course of neurodegenerative disease.

Another striking hotspot is the epidemiological link between common vaccinations and reduced dementia risk, which has introduced a prevention paradigm into the field. Large cohort studies and retrospective analyses have consistently found that older individuals who received routine vaccinations are less likely to develop dementia or AD years later (58–60). Similar associations have been observed with the tetanus, diphtheria and Tdap combination vaccine, the herpes zoster (shingles) vaccine, and pneumococcal vaccines, each conferring approximately 20–30% risk reductions in dementia incidence (42). Even vaccines not originally developed for AD have shown promise: treatment with Bacillus Calmette–Guérin (BCG) for bladder cancer was associated with ~20% reduced risk of Alzheimer’s and related dementias over 15 years (61, 62). These findings have sparked a new research direction examining how vaccine may confer neuroprotective effects. Several hypotheses are being explored. Vaccines might reduce the burden of specific infections that have been implicated in dementia thereby preventing infection-triggered neuroinflammation; alternatively, vaccines could induce long-term systemic immune changes that bolster brain resilience to degenerative processes (63, 64). This epidemiological line of inquiry is cross-pollinating with traditional neuropathological research, evidenced by recent keyword bursts like “influenza vaccination,” “infection” and even “Parkinson’s disease,” suggesting a growing appreciation that neurodegenerative diseases may be influenced by immune system exposures and whole-body health. Importantly, these studies also serve a public health message. They counter vaccine misinformation by indicating that, far from harming the aging brain, vaccine might actually preserve cognitive health.

A review pooling six cohort studies of patients with non-muscle-invasive bladder cancer reported that intravesical BCG was associated with a 26% lower incidence of AD, with exploratory subgroup signals suggesting stronger protection in adults >75 years and in women (65). A 2023 review similarly found a pooled HR ≈ 0.55 for dementia after BCG (66). Conversely, a 2024 meta-analysis concluded that the overall impact is minimal at best, with two non-overlapping primary analyses yielding HR 0.65 and HR 0.63 and persistent heterogeneity despite sensitivity tests (67). Taken together, current evidence suggests a possible protective association between intravesical BCG and subsequent dementia in bladder-cancer cohorts, but residual confounding, exposure misclassification, competing-risk issues, BCG-strain/dose differences, and generalizability beyond cancer populations limit causal inference. Mechanistically, BCG-induced trained immunity offers a biologically plausible pathway for long-lived modulation of neuroinflammatory tone in aging (17).

Several recent studies provide granularity on when and how often vaccines were given. In a prospective UK Biobank cohort, influenza vaccination was modelled as a time-varying exposure and showed a dose–response: participants averaging >0.8 influenza vaccinations per year had a lower dementia risk, and the association persisted after excluding events in the first 2–4 years to mitigate reverse causality (68). Quasi-experimental zoster vaccine studies also speak to timing: policy-driven rollouts and vaccine-type switches show risk reductions over 6–7 years post-vaccination, including stronger effects in women, consistent with a medium-term protective window (15). For pneumococcal vaccination, analyses anchored to the age 65–75 window report ~25–30% lower subsequent Alzheimer’s risk, suggesting that vaccinating in the younger-old decade may matter (69). A large Danish registry analysis found a small increased risk with influenza vaccination, likely reflecting residual confounding. Together, these data suggest that frequency, earlier administration within older age, and vaccine type may shape effect size and durability (70).

### 4.3 Future research directions

Building on these trends, future research in the CI/dementia and vaccine field is poised to become even more translational and preventive in orientation. One clear direction is the pursuit of next-generation vaccine strategies targeting multiple pathological mechanisms simultaneously. The limitations of single-target approaches have led to efforts at “second-generation” or even “multivalent” vaccines that could address amyloid, tau, and other contributing proteins in one platform (54, 55). For example, researchers are designing combination vaccines that include epitopes from both Aβ and tau, aiming to elicit a broad immune response against both hallmark lesions of AD. Alongside this, improving the quality of the immune response is a priority. Novel adjuvants and delivery systems are being tested to provoke a more robust, precisely tuned immunity without causing autoimmune side effects. Marciani et al. (52) reviewed how traditional aluminum-based adjuvants may be suboptimal for neurodegenerative targets and discussed advanced adjuvant formulations to induce a favorable T_H1-biased response that could enhance microglial clearance of pathology. Similarly, vaccine technology is rapidly evolving-the success of mRNA vaccine platforms in infectious disease has prompted investigations into mRNA-based vaccines for AD, which could be customized to produce specific therapeutic antibodies or antigens *in vivo*. Another future direction is earlier intervention. As biomarker research has made it feasible to detect AD changes in asymptomatic or mildly impaired individuals (71), there is growing interest in prophylactic vaccination before significant neurodegeneration occurs. Coupled with this is the need to address immunosenescence in future vaccine designs. Older adults’ immune systems respond less vigorously to vaccines (72), so research is focusing on how to boost responses in this demographic.

The intriguing findings from observational studies also chart a course for future work. Researchers will aim to unravel the causal mechanisms behind the protective associations of vaccines against dementia. Long-term prospective studies and eventually randomized controlled trials could test whether interventions like annual flu shots or shingles vaccines directly reduce cognitive decline, and through what biological pathways (73). This line of inquiry may reveal novel targets for dementia prevention. There is also room to explore whether vaccine strategies might benefit other neurodegenerative conditions beyond AD. The emergence of “Parkinson’s disease” as a keyword burst suggests that concepts from AD immunotherapy are being examined for synucleinopathies or that epidemiological links between vaccines and other dementias are of interest. Future trials are likely to test active immunotherapies in diverse dementias and even combined pathologies common in very old populations. Additionally, holistic and combination approaches will gain prominence. As several of the most-cited papers argue, combating dementia may require a multifactorial strategy. Vaccines could be combined with anti-inflammatory treatments, lifestyle interventions, or neuroprotective drugs to yield synergistic effects (52, 54, 55). For example, mitigating environmental risk factors like chronic infections or toxins, such as mercury (74) might enhance the efficacy of a vaccine by reducing ongoing neural damage. Clinicians and policymakers will also grapple with questions of cost-effectiveness and implementation. As indicated by the presence of cost-effectiveness analyses in recent clusters, the field recognizes that any successful dementia vaccine will need to be not only clinically effective but also deliverable to large at-risk populations in a financially feasible way. A study on the development of an AD, the vaccine must not only prove its effectiveness in clinical trials but also consider its global economic viability (75). In summary, the next phase of research will likely emphasize early, combination interventions and cross-disciplinary integration, moving from proof-of-concept immunology toward real-world disease modification and prevention.

### 4.4 Limitations

Despite yielding insightful trends, our study has several limitations. First, the bibliometric analysis is constrained by the scope of the databases and search strategy. We included only English-language publications; therefore, relevant work published in other languages have been omitted. Second, relying on WOS and PubMed indexing means that very recent publications might not yet be fully indexed or cited, potentially underrepresenting the newest developments. And PubMed Title/Abstract-only search may miss MeSH-indexed records without these term. Third, while bibliometric tools like VOSviewer and CiteSpace are powerful for mapping knowledge domains, their analyses have inherent methodological biases. We mitigated this with standard, validated parameters, but some nuanced research themes could have been overlooked or lumped together. Lastly, our quantitative analysis does not directly assess research quality or clinical impact. A high publication count or citation count in a given subtopic does not necessarily mean that those approaches have proven effective clinically. Likewise, the observational studies linking vaccines to lower dementia risk, though statistically robust, cannot establish causality. Despite these limitations, our bibliometric review provides a comprehensive overview of the trajectory and current landscape of research at the intersection of CI, dementia, and vaccines.

## 5 Conclusion

Over the past quarter-century, research at the intersection of vaccine and cognitive decline has evolved from exploratory β-amyloid immunization experiments into a robust, global endeavor that now embraces passive antibody therapies, multi-target vaccine engineering, and compelling population data linking routine adult immunisations to reduced dementia incidence. Our bibliometric synthesis underscores three converging imperatives for the next phase: designing safer, multi-epitope or mRNA platforms capable of countering amyloid-tau-inflammatory networks; initiating vaccination earlier in at-risk individuals while tailoring adjuvants and dosing to overcome immunosenescence; and rigorously dissecting how conventional vaccines modulate neuroinflammatory pathways to confer cognitive resilience. Addressing these priorities, while embedding cost-effectiveness and equity considerations, is essential to transform immunization from a hopeful concept into a cornerstone of dementia prevention and disease modification.

## Data Availability

The raw data supporting the conclusions of this article will be made available by the authors, without undue reservation.

## Footnotes

^1^ https://donghaoren.org/charticulator/index.html
